# Class effect of beta-blockers in survivors of ST-elevation myocardial infarction: A nationwide cohort study using an insurance claims database

**DOI:** 10.1038/srep13692

**Published:** 2015-09-02

**Authors:** Ting-Tse Lin, K. Arnold Chan, Ho-Min Chen, Chao-Lun Lai, Mei-Shu Lai

**Affiliations:** 1 Department of Internal Medicine, National Taiwan University Hospital Hsin-Chu Branch, Hsin-Chu, Taiwan; 2 Department of Medical Research, National Taiwan University Hospital, Taipei, Taiwan; 3 Graduate Institute of Oncology, National Taiwan University College of Medicine, Taipei, Taiwan; 4 Center for Comparative Effectiveness Research, National Center of Excellence for Clinical Trial and Research, National Taiwan University Hospital, Taipei, Taiwan; 5 Department of Internal Medicine, National Taiwan University College of Medicine, Taipei, Taiwan; 6 Institute of Epidemiology and Preventive Medicine, College of Public Health, National Taiwan University, Taipei, Taiwan

## Abstract

Beta-blockers can help reduce mortality following acute myocardial infarction (MI); however, whether beta-blockers exert a class effect remains controversial. This study identified all patients with first ST-elevation MI for the period of 2003 to 2010 from the National Health Insurance claims database, Taiwan. We compared patients prescribed carvedilol, bisoprolol, and propranolol. Study outcomes included all-cause death, cardiovascular death, and recurrence of MI. The propensity scores were constructed using multinomial logistic regression to model the receipt of different beta-blockers. Treating carvedilol group as a reference, we employed a simultaneous three-group comparison approach using the Cox regression model with adjustment for the propensity scores to compare the relative risks of various outcomes. Among the 16836 patients, 7591 were prescribed carvedilol, 5934 bisoprolol, and 3311 propranolol. Mean follow-up time was one year. After accounting for baseline differences, patients treated with bisoprolol (HR 0.87, 95% CI 0.72–1.05, p = 0.14) or propranolol (HR 1.07, 95% CI 0.84–1.36, p = 0.58) had a similar risk of all-cause death in comparison with carvedilol. No significant differences were observed among three beta-blocker groups with regard to the risks of cardiovascular death and recurrence of MI. Our results suggest that beta-blockers exert a possible class effect in the treatment of acute MI.

Beta-blocker therapy is the standard treatment for ST-elevation myocardial infarction (STEMI). The 2013 American College of Cardiology Foundation/American Heart Association Guidelines for the Management of STEMI state that STEMI should be treated with oral beta-blockers in patients without contraindications (Class I indication)^1^. Benefits of beta-blockers for patients with acute myocardial infarction (MI) include anti-ischemic, antihypertensive, antiarrhythmic, and antithrombotic effects^2^. Most evidence supporting the benefits of beta-blockers has been obtained from randomized trials pre-dating the advent of modern reperfusion therapy and pharmacotherapy^3^,^4^,^5^,^6^. In the era of percutaneous coronary intervention (PCI), several prospective cohort studies have also indicated that treatment with beta-blockers is associated with reduced mortality in patients suffering from acute MI^7^,^8^,^9^,^10^

Most physicians assume that all beta-blockers exert a class effect; therefore, there is considerable variation in the type of beta-blocker prescribed to treat acute MI^11^. However, given the differences in pharmacologic properties among available beta-blockers, this assumption is questionable^12^. The current study investigated long-term outcomes of STEMI patients treated with different beta-blockers (carvedilol, bisoprolol, and propranolol). Subjects were identified from the National Health Insurance (NHI) claims database in Taiwan.

## Results

### Characteristics of study subjects

**Figure 1:**
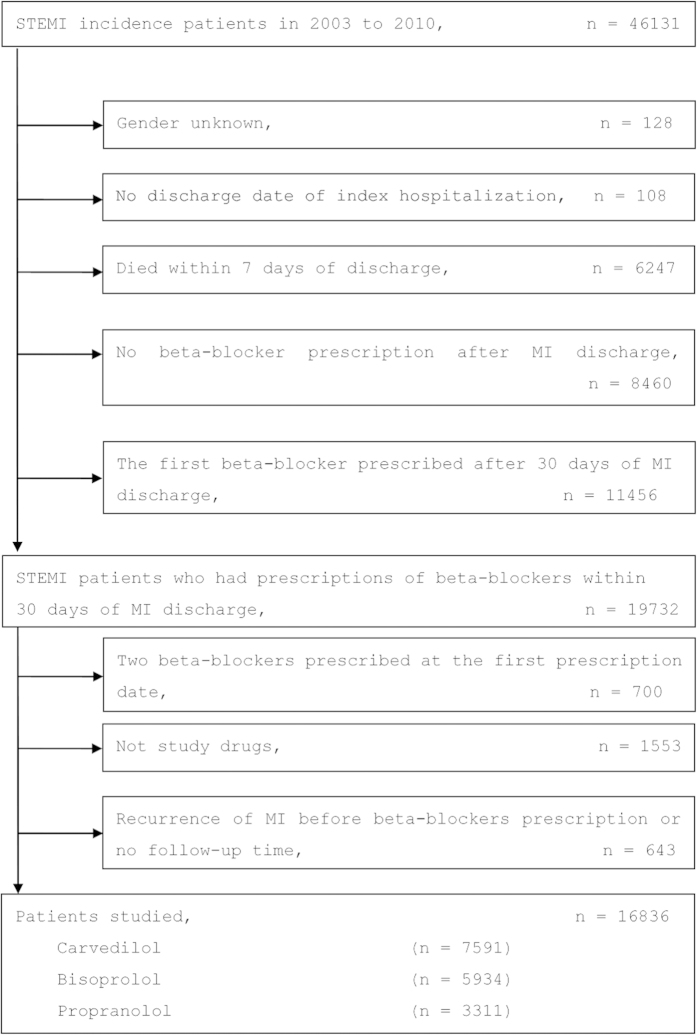
A flowchart illustrating the process of patient identification. Abbreviations: MI, myocardial infarction; STEMI, ST-elevation myocardial infarction .

We identified a total of 16836 patients that met selection criteria in the NHI claims database for the period covering January 2003 to December 2010. Among them, 7591 (45%) patients were prescribed carvedilol, 5934 (35%) were prescribed bisoprolol, and 3311 (20%) were prescribed propranolol (Fig. 1).

The majority of the study population was considered to be at intermediate risk (i.e. 10%–20% 10-year risk of coronary heart disease according to the Framingham Risk Score)^13^. In addition, most patients were male, and the median age of subjects was 61 years. Patients prescribed bisoprolol were more likely to also have a prescription for clopidogrel or statin than patients belonging to the carvedilol group, but less likely to have prescriptions for loop diuretics, spironolactone, or amiodarone. Compared with the carvedilol group, patients prescribed propranolol were younger, less likely to suffer from congestive heart failure (CHF) or diabetes with chronic complications, and less likely to have a prescription for clopidogrel, ARBs, loop diuretics, spironolactone, statins, amiodarone, or insulin. Totally, 62.2% patients received coronary angiography during the index hospitalization; 1.9% received CABG; and 7.0% received t-PA. Comparing treatment groups, patients prescribed bisoprolol were more likely to receive coronary angiography. Conversely, patients treated with propranolol were less likely than the carvedilol group to receive coronary angiography but more likely to receive t-PA (Table 1).

### Main results

The mean follow-up time was 1.0 year, ending on Dec. 31, 2011. Overall, the accumulated incidence of all-cause death was 3.7%; for cardiovascular death, it was 1.8%; and the recurrence of MI was 7.3% (Table 2). In the unadjusted Cox model, treatment with bisopolol was associated with a lower risk of all-cause death (unadjusted hazard ratio [HR] 0.62, 95% confidence interval [CI] 0.52–0.74, p < 0.001) and cardiovascular death (unadjusted HR 0.64, 95% CI 0.50–0.82, P < 0.001) than treatment with carvedilol. Treatment with propranolol also presented a lower risk of cardiovascular death (unadjusted HR 0.66, 95% CI 0.46–0.95, p = 0.024) than the carvedilol group (Table 3).

However, using a simultaneous three-group comparison approach and adjusting for age, sex, and the propensity scores, we found no difference between the bisoprolol group and the carvedilol group with regard to risks of all-cause death (adjusted HR 0.87, 95% CI 0.72–1.05, p = 0.14), cardiovascular death (adjusted HR 0.87, 95% CI 0.68–1.13, p = 0.30), and recurrence of MI (adjusted HR 0.97, 95% CI 0.85–1.10, p = 0.63). Similarly, patients treated with propranolol and patients treated with carvedilol presented similar risk levels with regard to all-cause death (adjusted HR 1.07, 95% CI 0.84–1.36, p = 0.58), cardiovascular death (adjusted HR 0.92, 95% CI 0.64–1.32, p = 0.64) and recurrence of MI (adjusted HR 1.14, 95% CI 0.97–1.33, p = 0.12). The analyses using the pairwise contrast approach (either with adjustment for the propensity scores or stratification on the propensity score quintiles) yielded similar results (Table 3).

### Subgroup analyses

**Figure 2:**
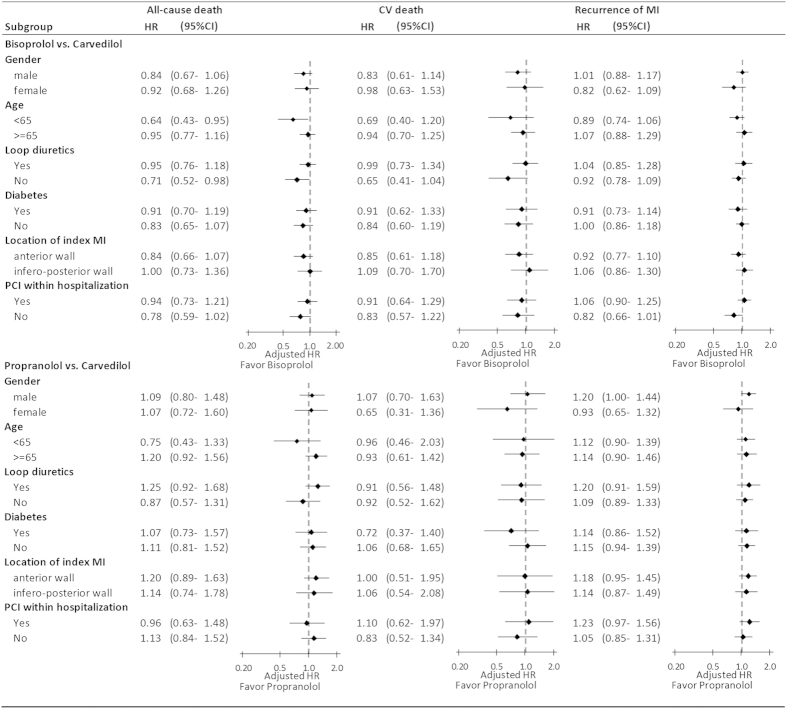
Relative risks of various clinical outcomes associated with the three beta-blockers, stratified according to subgroups. Abbreviations: CI, confidence interval; CV, cardiovascular; HR, hazard ratio; MI, myocardial infarction; PCI, percutaneous coronary intervention.

The lack of difference in risks of clinical outcomes between the three beta-blockers was consisted in most of the prespecified subgroups including gender, age, use/non-use of loop diuretics, diabetes status, location of the index MI and receiving PCI or not during the index hospitalization. However, we found an association between bisoprolol and a reduced risk of all-cause death among younger (<65 years old) patients (adjusted HR 0.64, 95% CI 0.43–0.95) and patients not receiving loop diuretics (adjusted HR 0.71, 95% CI 0.52–0.98) in comparison with carvedilol group (Fig. 2).

### Sensitivity analyses

In the two sensitivity analyses, the exclusion criterion of beta-blockers prescribed more than 30 days after discharge was replaced with 14 days and 42 days, respectively. All the results remained unchanged in both sensitivity analyses (supplementary Table 1 and Table 2).

## Discussion

For this study, we investigated three beta-blockers that are most commonly prescribed in Taiwan (Supplementary Table 3). Carvedilol, a non-selective beta-blocker with alpha-blocker activity, has pleiotropic effects including anti-oxidation and vasodilation^14^. Bisoprolol is a selective beta-1 receptor blocker without intrinsic sympathomimetic activity (ISA). In contrast, propranolol is a non-selective beta-blocker with a shorter half-life. The primary purpose of this study was to examine whether beta-blockers exert a class effect in terms of reducing mortality in post-MI patients. Our unadjusted results indicate that bisoprolol and propranolol may be more effective in this regard. Nevertheless, following adjustment for baseline characteristics, no differences related to risk of all-cause death, cardiovascular death, or recurrence of MI were observed between carvedilol, bisoprolol, and propranolol groups.

Prior to the era of reperfusion and thrombolytics, a meta-regression analysis had found that treating post-MI patients with beta-blockers at the time of discharge improved survival by approximately 25%^15^. Furthermore, in the Carvedilol Post-Infarct Survival Control in LV Dysfunction (CAPRICORN) study and other studies on acute MI patients, carvedilol was also shown to reduce overall mortality^16^,^17^. Similar reductions in mortality were reported in trials investigating the effect of propranolol among post-MI patients^5^,^18^,^19^. Bisoprolol was shown to reduce all-cause mortality in patients with heart failure. However, no trial has been conducted to evaluate treatment efficacy of bisoprolol in patients with acute MI^20^. Although current guidelines support the use of beta-blockers in all eligible MI survivors^1^,^6^, a number of head-to-head comparisons between various types of beta-blockers have yielded conflicting conclusions. For example, one open-label study comparing carvedilol with atenolol showed no significant differences in primary endpoints after a median follow-up time of 1.6 years^21^. Another study of 313 patients that compared metoprolol with carvedilol over a mean duration of 13.4 months also found no differences with regard to different clinical endpoints^22^. However, one meta-analysis indicated that the efficacy of carvedilol in reducing all-cause mortality significantly exceeded that of selective beta-1 blockers (atenolol, bisoprolol, metoprolol, and nebivolol) in MI patients^23^. Higher mortality rates were observed in patients received metoprolol compared with those that received atenolol or acebutolol^24^. Furthermore, patients prescribed propranolol at the time of discharge presented a slightly higher mortality rate than patients prescribed metoprolol or atenolol^25^. These conflicting findings suggest that the assumption that beta-blockers exert a class effect for secondary prevention after acute MI is questionable. Besides, a few researchers proposed that the ancillary properties (i.e., ISA, beta 1-selectivity, membrane stabilizing activity, and lipophilicity) of different beta-blockers might have influence on their clinical efficacy^26^.

The all-cause death rate in our study was 3.7%, which is similar to the results obtained from other studies conducted among post-MI patients treated with beta-blockers in the contemporary PCI era (approximately 3–4% over a one-year follow-up)^9^,^10^,^27^. The crude results of our study seem to indicate that the use of bisoprolol or propranolol is associated with a reduction in all-cause death and cardiovascular death compared with use of carvedilol. However, significant differences were observed in baseline demographics and clinical characteristics of patients treated with different beta-blockers. Overall, patients prescribed carvedilol were more likely to have CHF and used more medications (such as loop diuretics, spironolactone, and amiodarone) compared with patients in the bisorpolol and propranolol groups. After adjusting for baseline characteristics, no significant differences were observed between the three beta-blockers with regard to effectiveness in reducing mortality. All the three different methods of statistical analysis yielded the same results, demonstrating the robustness of our findings. Our results imply a class effect of beta-blockers in the treatment of patients suffering from STEMI and support the concept that specific beta-blockers have little influence on mortality^21^,^22^,^25^

CHF is a common complication of acute MI and presents in 20–30% of MI survivors^28^,^29^,^30^,^31^. In the literature, carvedilol, bisoprolol, and metoprolol succinate are the preferred drugs for patients with a reduced left ventricle ejection fraction (LVEF)^32^,^33^. Although there was no individual LVEF in our database, we chose the use of loop diuretics at discharge as a surrogate for presence of CHF after the index MI. In our study, the proportion of patients prescribed loop diuretics at discharge was 37%, which was similar to the prevalence rate of CHF in several registries and clinical trials concerning acute MI patients^28^,^29^,^30^,^31^. In our subgroup analysis, we still noted no differences between the three study drugs with regard to the risks of different clinical outcomes among patients prescribed loop diuretics at discharge.

### Study limitations

A number of limitations in the present study must be acknowledged. Firstly, in most previous studies, metoprolol was examined with regard to its effects in the secondary prevention in post-MI patients. However, the patient population using metorpolol in Taiwan was too small to be included in our analysis (Supplementary Table 3). Secondly, we were unable to conduct a randomized, controlled trial; therefore, our results may have been affected by defects inherent to non-randomized comparisons. These include selection bias and an uneven distribution of risk factors. To address these issues, we conducted several statistical methods with utilization of propensity scores to control for detected differences between groups. Thirdly, although we attempted to control for the majority of known risk factors, it is possible that some factors were not properly accounted for. For example, we were unable to access data related to LVEF or drug adherence, as this information is not available in the NHI database. We tried to control for left ventricular dysfunction using prescriptions of loop diuretics as a proxy for left ventricular systolic dysfunction. However, the results may vary according to different LVEF. Besides, the inability to evaluate patient compliance in taking the prescribed beta-blockers means that their true effects may have been under-represented. Fourthly, our follow-up duration (one-year) was shorter than that of many large trials and registries (i.e. a median follow-up time of between two and four years^10^,^27^,^34^). The similar outcomes associated with the three drugs investigated may therefore be due to a lack of power and a follow-up of insufficient duration. Finally, we did not adjust for in-hospital administration of beta-blockers; therefore, we are unable to evaluate the benefits of early beta-blocker usage after acute MI, as reported in the Effect of Metoprolol in Cardioprotection During an Acute Myocardial Infarction (METOCARD-CNIC) trial^35^.

## Conclusions

In the real-world population-based setting of Taiwan, carvedilol, bisoprolol and propranolol possessed similar clinical effectiveness in STEMI survivors. In spite of the exclusion of metoprolol, one of the most commonly used cardioselective beta-blockers in other countries, our results are still suggestive of a possible class effect of beta-blockers.

## Materials and Methods

### Sources of data

The NHI program has provided compulsory universal health insurance in Taiwan since 1995. More than 98% of the entire Taiwanese population of 23 million is covered by the program. All medical institutions contracted with the NHI must submit standard computerized claims in order to obtain reimbursement. As a result, the NHI claims database contains a nearly complete history of diagnoses (classified according to the International Classification of Diseases Ninth Revision Clinical Modification [ICD-9-CM] codes) as well as records for medical procedures and drugs dispensed. In this study, we extracted all of the records of the study subjects from the NHI claims database and linked to the National Death Registry (NDR) for mortality outcomes using the identification number of each patient. To comply with data privacy regulations, personal identities were encrypted and all data were analyzed in a de-identified manner. The protocol for this study was approved by the Institutional Review Board of National Taiwan University Hospital.

### Study population

The NHI claims database was investigated for the period covering 1998 to 2011. We identified all patients who were above 18 years old and had their first hospitalization for STEMI (ICD-9-CM codes, 410.1–410.6 and 410.8) between January 2003 and December 2010. Several exclusion criteria were applied to increase the reliability and validity of our results. Specifically, patients were excluded if: (1) they had previously been hospitalized for acute MI between 1998 and 2002, (2) their gender was unknown, (3) the discharge date was missed in the index hospitalization, (4) death occurred within 7 days of discharge, (5) MI recurred prior to the initial prescription of beta-blockers, (6) they were prescribed two beta-blockers at the same time, (7) they were prescribed beta-blockers other than the drugs specified in the study, (8) beta-blockers were not prescribed following discharge throughout the study period or were prescribed 30 days after discharge. A flowchart of the process used to identify study subjects is presented in Fig. 1.

### Drug use, covariates, and outcomes

Three most commonly prescribed beta-blockers, carvedilol, bisoprolol, and propranolol, in post-MI population in Taiwan were included in the current study (Supplementary Table 3). Patients were classified as users of carvedilol, bisoprolol, or propranolol based on the first prescription filled for a beta-blocker within 30 days post discharge. The date of the first beta-blocker prescription was operationally defined as the index date. In addition to recording gender and age on index date, we evaluated comorbidities based on NHI claims data filed within the twelve-month baseline period prior to the index date. Specifically, we obtained information for several comorbidities, including CHF, cerebrovascular disease, chronic pulmonary disease, dementia, diabetes, liver disease, peptic ulcer disease, and renal disease, in accordance with Charlson comorbidity measurements^36^. The presence of a comorbid condition was defined as the specific diagnosis codes recorded in claims data for at least two times on different days within the twelve-month baseline period. Individual comorbid conditions were evaluated and coded as binary variables. Medications that were prescribed at discharge during the index hospitalization, including aspirin, clopidogrel, warfarin, calcium channel blockers (CCBs), angiotensin-converting-enzyme inhibitors (ACEIs), angiotensin receptor blockers (ARBs), loop diuretics, spironolactone, statins, amiodarone, oral anti-diabetic drugs (OADs), and insulin were identified. For health care utilizations, we recorded the numbers of out-patient and in-patient services each patient used within the twelve-month baseline period prior to the index date. During the index hospitalization, cardiac procedures including coronary angiography, coronary artery bypass graft (CABG), and the use of tissue plasminogen activator (t-PA) were also evaluated.

Due to the availability of NHI and NDR data, the mortality of study subjects and other clinical outcomes were assessed through to December 31, 2011. Subjects were censored if they switched to another beta-blocker, in cases where study drugs were discontinued for more than 30 days, or at the end of the follow-up period. Study outcomes included all-cause death, cardiovascular death (ICD-9-CM codes, 401–449 as the cause of death), and recurrence of MI (ICD-9-CM codes, 401.0–410.9 in discharge diagnoses).

### Statistical analyses

To enable a comparison of baseline characteristics between the three beta-blockers groups, the χ^2^ test, the two sample t-test and the Mann-Whitney U test were used with the carvedilol group as the reference. We used standardized difference to measure covariate balance, whereby an absolute standardized difference of greater than 10% represented meaningful imbalance.

Because of the heterogeneity of three groups, the propensity scores were constructed using multinomial logistic regression to model the receipt of different beta-blockers as a function of baseline patient characteristics^37^,^38^. All the background characteristics listed in Table 1, such as age, gender, comorbidities, medications, medical utilizations and procedures received during the index hospitalization, were included in the multinomial logistic regression model during construction of the propensity scores. As propensity score – matching usually results in marked reduction in sample size^39^,^40^, a simultaneous three-group comparison approach using the Cox regression model with adjustment for age, sex, and the propensity scores was used to compare the relative risks of various outcomes associated with the three beta-blockers^37^,^41^. Carvedilol, the beta-blocker most frequently used by MI patients in Taiwan, was selected *a priori* as the reference category. We also applied the pairwise contrast methods using the Cox regression model with adjustment for the propensity scores and the Cox regression model stratified on quintiles of the propensity scores. Subgroup analysis was also performed using the simultaneous three-group comparison approach, and the subgroups included gender, age, use/non-use of loop diuretics, diabetes status, location of the index MI and receiving PCI or not during the index hospitalization.

In the primary analysis, patients who were prescribed beta-blockers after 30 days of discharge were excluded. Since the 30-day criterion was arbitrary, we also performed two sensitivity analysis with 14-day and 42-day exclusion criteria to test the robustness of our study design and results.

All analyses were performed using SAS 9.2 software (SAS Institute Inc., Cary, North Carolina). A p-value of <0.05 was considered statistically significant.

## Additional Information

**How to cite this article**: Lin, T.-T. *et al.* Class effect of beta-blockers in survivors of ST-elevation myocardial infarction: A nationwide cohort study using an insurance claims database. *Sci. Rep.* **5**, 13692; doi: 10.1038/srep13692 (2015).

## Supplementary Material

Supplementary Information

## Figures and Tables

**Table 1:** Demographic and clinical characteristics of study subjects.

			**Bisoprolol**			**Propranolol**		
**Variable**	**Total**	**Carvedilol**		p*	SD*		**p**†	**SD**†
Patients (n)	16836	7591	5934			3311		
Female (%)	20.8	21.7	19.7	0.005	0.05	20.8	0.29	0.02
Age (years, Mean)	61.3	62.1	60.7	<0.001	0.10	60.6	<0.001	0.11
Comorbidities (%)								
Congestive Heart failure	5.8	6.9	5.2	<0.001	0.07	4.3	<0.001	0.12
Cerebrovascular disease	9.1	9.7	8.8	0.06	0.03	8.2	0.010	0.06
Chronic pulmonary disease	8.1	8.6	7.7	0.07	0.03	7.7	0.12	0.03
Dementia	1.3	1.4	1.1	0.15	0.03	1.3	0.91	<0.01
Diabetes without chronic complication	23.6	24.7	23.8	0.26	0.02	20.9	<0.001	0.09
Diabetes with chronic complication	7.1	8.4	6.1	<0.001	0.09	5.5	<0.001	0.12
Liver disease	5.2	5.2	5.0	0.58	0.01	5.5	0.55	0.01
Peptic ulcer disease	9.7	9.6	9.6	0.97	<0.01	10.2	0.31	0.02
Renal disease	4.9	5.6	4.4	0.002	0.06	4.1	0.001	0.07
Prescriptions at discharge (%)								
Aspirin	96.9	96.7	97.5	0.009	0.05	96.3	0.34	0.02
Clopidogrel	88.0	88.7	92.7	<0.001	0.14	78.1	<0.001	0.29
Warfarin	3.0	3.6	2.3	<0.001	0.08	2.7	0.028	0.05
CCBs	23.8	23.1	24.3	0.12	0.03	24.6	0.08	0.04
ACEIs	73.9	75.6	72.6	<0.001	0.07	72.4	<0.001	0.07
ARBs	19.8	20.1	23.3	<0.001	0.08	13.1	<0.001	0.19
Loop diuretics	37.0	44.5	33.0	<0.001	0.24	26.9	<0.001	0.37
Spironolactone	10.6	13.6	9.6	<0.001	0.13	5.5	<0.001	0.28
Statins	55.7	53.9	63.4	<0.001	0.19	46.0	<0.001	0.16
Amiodarone	13.6	16.2	12.4	<0.001	0.11	10.0	<0.001	0.18
OADs	27.0	28.2	27.2	0.22	0.02	23.8	<0.001	0.10
Insulin	20.1	22.4	18.7	<0.001	0.09	17.1	<0.001	0.13
Medical utilizations (median)								
Number of OPD visits	16	16	16	0.95	<0.01	16	0.69	0.03
Number of hospitalizations	0	0	0	0.93	0.05	0	0.10	0.03
Cardiac procedures during index hospitalization (%)								
Coronary angiography	62.2	60.5	72.1	<0.001	0.25	48.6	<0.001	0.25
CABG	1.9	2.2	1.9	0.34	0.02	1.4	0.004	0.06
t-PA	7.0	6.2	5.4	0.06	0.03	11.8	<0.001	0.20

Abbreviations: ACEI, angiotensin-converting-enzyme inhibitor; ARB, angiotensin receptor blocker; CABG, coronary artery bypass graft; CCB, calcium channel blocker; OAD, oral anti-diabetic drug; OPD, out-patient department; SD, standardized difference; t-PA, tissue plasminogen activator.

^*^Bisoprolol vs. Carvedilol.

^†^Propranolol vs. Carvedilol.

**Table 2:** Clinical outcomes associated with the three beta-blocker groups.

	**Total**	**Carvedilol**	**Bisoprolol**	**Propranolol**
n	16836	7591	5934	3311
Follow-up time (years)				
Mean (SD)	1.0 (1.3)	1.0 (1.3)	1.3 (1.4)	0.6 (1.0)
All-cause death, n (%)	624 (3.7%)	345 (4.5%)	193 (33%)	86 (2.6%)
CV death, n (%)	309 (1.8%)	174 (2.3%)	99 (1.7%)	36 (1.1%)
Recurrence of MI, n (%)	1229 (7.3%)	564 (7.4%)	442 (7.5%)	223 (6.7%)

Abbreviations: CV, cardiovascular; MI, myocardial infarction; SD, standard deviation.

**Table 3:** Relative risks of various clinical outcomes associated with the three beta-blocker groups.

	**All-cause death**			**CV death**			**Recurrence of MI**		
Crude results									
Drug	HR	95% CI	p	HR	95% CI	p	HR	95% CI	p
Carvedilol	1			1			1		
Bisoprolol	0.62	(0.52–0.74)	<0.001	0.64	(0.50–0.82)	<0.001	0.92	(0.81–1.04)	0.18
Propranolol	0.81	(0.64–1.03)	0.08	0.66	(0.46–0.95)	0.024	1.12	(0.96–1.31)	0.16
Simultaneous three-group comparison with adjustment for the propensity scores*									
Carvedilol	1			1			1		
Bisoprolol	0.87	(0.72–1.05)	0.14	0.87	(0.68–1.13)	0.30	0.97	(0.85–1.10)	0.63
Propranolol	1.07	(0.84–1.36)	0.58	0.92	(0.64–1.32)	0.64	1.14	(0.97–1.33)	0.12
Pairwise contrast with adjustment for the propensity scores†									
Carvedilol	1			1			1		
Bisoprolol	0.88	(0.73–1.06)	0.17	0.88	(0.68–1.13)	0.31	0.98	(0.86–1.12)	0.76
Propranolol	1.06	(0.83–1.36)	0.62	0.90	(0.62–1.31)	0.58	1.12	(0.95–1.31)	0.18
Pairwise contrast with stratification on quintiles of the propensity scores‡									
Carvedilol	1			1			1		
Bisoprolol	0.86	(0.72–1.04)	0.11	0.87	(0.67–1.13)	0.29	0.98	(0.86–1.12)	0.77
Propranolol	1.04	(0.81–1.33)	0.77	0.89	(0.61–1.29)	0.53	1.13	(0.96–1.33)	0.14

^*^Simultaneous three-group comparison using the Cox proportional hazards model with adjustment for age, sex, and the propensity scores in which carvedilol is treated as the reference group.

^†^Repeated pairwise comparison using the Cox proportional hazards model with adjustment for age, sex, and the propensity scores in which carvedilol is treated as the reference group.

^‡^Repeated pairwise comparison using the Cox proportional hazards model with adjustment for age, sex, and stratified on quintiles of the propensity scores in which carvedilol is treated as the reference group.
