# Supplementary material for: Class effect of beta-blockers in survivors of ST-elevation myocardial infarction: A nationwide cohort study using an insurance claims database
Source: Sci Rep. 2015 Sep 2;5:13692. doi: 10.1038/srep13692 (PMC4642576; doi:10.1038/srep13692)
Supplement: Supplementary Information [file srep13692-s1.doc]

**Class effect of beta-blockers in survivors of ST-elevation myocardial infarction: A nationwide cohort study using an insurance claims database**

Ting-Tse Lin^1^, K. Arnold Chan^2,3^, Ho-Min Chen^4^, Chao-Lun Lai^1,5,6^, Mei-Shu Lai^4,6^

^1^Department of Internal Medicine, National Taiwan University Hospital Hsin-Chu Branch, Hsin-Chu, Taiwan

^2^Department of Medical Research, National Taiwan University Hospital, Taipei, Taiwan

^3^Graduate Institute of Oncology, National Taiwan University College of Medicine, Taipei, Taiwan

^4^Center for Comparative Effectiveness Research, National Center of Excellence for Clinical Trial and Research, National Taiwan University Hospital, Taipei, Taiwan

^5^Department of Internal Medicine, National Taiwan University College of Medicine, Taipei, Taiwan

^6^Institute of Epidemiology and Preventive Medicine, College of Public Health, National Taiwan University, Taipei, Taiwan

**Running Head: beta-blockers in acute myocardial infarction**

Corresponding author:

Chao-Lun Lai, M.D., Ph.D., Department of Internal Medicine, National Taiwan University Hospital Hsin-Chu Branch, Hsin-Chu, Taiwan. Tel: +886-3-5326151; Fax: +886-3-5353313; E-mail: chaolunlai@ntu.edu.tw

Number of supplementary tables: 3

***Supplementary Table 1. Relative risks of various clinical outcomes associated with the three beta-blocker groups*** *(using 14-day exclusion criterion, n*=15465)

|  | | **All-cause death** | | | | | **CV death** | | | | | **Recurrence of MI** | | | | | |
| --- | --- | --- | --- | --- | --- | --- | --- | --- | --- | --- | --- | --- | --- | --- | --- | --- | --- |
| ***Crude results*** | | | | | | | | | | | | | | | | | |
| **Drug** | **HR** | | **95% CI** | | **p** | | **HR** | **95% CI** | | **p** | | **HR** | **95% CI** | | **p** | |  |
| Carvedilol | 1 | |  | |  | | 1 |  | |  | | 1 |  | |  | |  |
| Bisoprolol | 0.61 | | ( 0.51-0.74 ) | | <0.001 | | 0.60 | ( 0.46-0.77 ) | | <0.001 | | 0.91 | ( 0.80-1.04 ) | | 0.17 | |  |
| Propranolol | 0.80 | | ( 0.63-1.02 ) | | 0.08 | | 0.69 | ( 0.48-1.00 ) | | 0.047 | | 1.13 | ( 0.96-1.32 ) | | 0.14 | |  |
| ***Simultaneous three-group comparison with adjustment for the propensity scores**** | | | | | | | | | | | | | | | | | |
| **Drug** | **HR** | | **95% CI** | | **p** | | **HR** | **95% CI** | | **p** | | **HR** | **95% CI** | | **p** | |  |
| Carvedilol | | 1 | |  | |  | 1 | |  | |  | 1 | |  | |  | |
| Bisoprolol | | 0.87 | | ( 0.72-1.05 ) | | 0.14 | 0.85 | | ( 0.65-1.11 ) | | 0.23 | 0.98 | | ( 0.86-1.12 ) | | 0.76 | |
| Propranolol | | 1.05 | | ( 0.81-1.35 ) | | 0.72 | 0.95 | | ( 0.66-1.38 ) | | 0.80 | 1.13 | | ( 0.96-1.34 ) | | 0.14 | |
| ***Pairwise contrast with adjustment for the propensity scores***† | | | | | | | | | | | | | | | | | |
| **Drug** | **HR** | | **95% CI** | | **p** | | **HR** | **95% CI** | | **p** | | **HR** | **95% CI** | | **p** | |  |
| Carvedilol | | 1 | |  | |  | 1 | |  | |  | 1 | |  | |  | |
| Bisoprolol | | 0.87 | | ( 0.72-1.05 ) | | 0.15 | 0.85 | | ( 0.65-1.11 ) | | 0.23 | 0.99 | | ( 0.86-1.13 ) | | 0.83 | |
| Propranolol | | 1.04 | | ( 0.80-1.34 ) | | 0.79 | 0.93 | | ( 0.64-1.34 ) | | 0.68 | 1.10 | | ( 0.93-1.30 ) | | 0.26 | |
| ***Pairwise contrast with stratification on quintiles of the propensity scores***‡ | | | | | | | | | | | | | | | | | |
| **Drug** | **HR** | | **95% CI** | | **p** | | **HR** | **95% CI** | | **p** | | **HR** | **95% CI** | | **p** | |  |
| Carvedilol | | 1 | |  | |  | 1 | |  | |  | 1 | |  | |  | |
| Bisoprolol | | 0.85 | | ( 0.70-1.03 ) | | 0.09 | 0.83 | | ( 0.63-1.09 ) | | 0.18 | 0.99 | | ( 0.87-1.13 ) | | 0.87 | |
| Propranolol | | 1.01 | | ( 0.78-1.31 ) | | 0.93 | 0.91 | | ( 0.63-1.33 ) | | 0.62 | 1.11 | | ( 0.94-1.32 ) | | 0.22 | |

*** Simultaneous three-group comparison using the Cox proportional hazards model with adjustment for age, sex, and the propensity scores in which carvedilol is treated as the reference group.

† Repeated pairwise comparison using the Cox proportional hazards model with adjustment for age, sex, and the propensity scores in which carvedilol is treated as the reference group.

‡ Repeated pairwise comparison using the Cox proportional hazards model with adjustment for age, sex, and stratified on quintiles of the propensity scores in which carvedilol is treated as the reference group.

***Supplementary Table 2. Relative risks of various clinical outcomes associated with the three beta-blocker groups*** *(using 42-day exclusion criterion, n*=18323)

|  | | **All-cause death** | | | | | **CV death** | | | | | **Recurrence of MI** | | | | | |
| --- | --- | --- | --- | --- | --- | --- | --- | --- | --- | --- | --- | --- | --- | --- | --- | --- | --- |
| ***Crude results*** | | | | | | | | | | | | | | | | | |
| **Drug** | **HR** | | **95% CI** | | **p** | | **HR** | **95% CI** | | **p** | | **HR** | **95% CI** | | **p** | |  |
| Carvedilol | 1 | |  | |  | | 1 |  | |  | | 1 |  | |  | |  |
| Bisoprolol | 0.61 | | ( 0.52-0.72 ) | | <0.001 | | 0.63 | ( 0.50-0.79 ) | | <0.001 | | 0.91 | ( 0.81-1.03 ) | | 0.13 | |  |
| Propranolol | 0.91 | | ( 0.73-1.12 ) | | 0.37 | | 0.79 | ( 0.58-1.08 ) | | 0.13 | | 1.13 | ( 0.97-1.31 ) | | 0.11 | |  |
| ***Simultaneous three-group comparison with adjustment for the propensity scores**** | | | | | | | | | | | | | | | | | |
| **Drug** | **HR** | | **95% CI** | | **p** | | **HR** | **95% CI** | | **p** | | **HR** | **95% CI** | | **p** | |  |
| Carvedilol | | 1 | |  | |  | 1 | |  | |  | 1 | |  | |  | |
| Bisoprolol | | 0.86 | | ( 0.72-1.03 ) | | 0.10 | 0.87 | | ( 0.68-1.11 ) | | 0.27 | 0.96 | | ( 0.85-1.09 ) | | 0.55 | |
| Propranolol | | 1.16 | | ( 0.94-1.45 ) | | 0.17 | 1.07 | | ( 0.77-1.47 ) | | 0.70 | 1.15 | | ( 0.99-1.34 ) | | 0.07 | |
| ***Pairwise contrast with adjustment for the propensity scores***† | | | | | | | | | | | | | | | | | |
| **Drug** | **HR** | | **95% CI** | | **p** | | **HR** | **95% CI** | | **p** | | **HR** | **95% CI** | | **p** | |  |
| Carvedilol | | 1 | |  | |  | 1 | |  | |  | 1 | |  | |  | |
| Bisoprolol | | 0.87 | | ( 0.73-1.03 ) | | 0.11 | 0.88 | | ( 0.68-1.12 ) | | 0.28 | 0.97 | | ( 0.86-1.10 ) | | 0.64 | |
| Propranolol | | 1.15 | | ( 0.92-1.43 ) | | 0.22 | 1.03 | | ( 0.75-1.42 ) | | 0.86 | 1.14 | | ( 0.97-1.33 ) | | 0.11 | |
| ***Pairwise contrast with stratification on quintiles of the propensity scores***‡ | | | | | | | | | | | | | | | | | |
| **Drug** | **HR** | | **95% CI** | | **p** | | **HR** | **95% CI** | | **p** | | **HR** | **95% CI** | | **p** | |  |
| Carvedilol | | 1 | |  | |  | 1 | |  | |  | 1 | |  | |  | |
| Bisoprolol | | 0.86 | | ( 0.72-1.02 ) | | 0.09 | 0.87 | | ( 0.68-1.11 ) | | 0.27 | 0.97 | | ( 0.86-1.10 ) | | 0.66 | |
| Propranolol | | 1.11 | | ( 0.89-1.38 ) | | 0.37 | 1.00 | | ( 0.72-1.38 ) | | 0.99 | 1.15 | | ( 0.98-1.34 ) | | 0.09 | |

*** Simultaneous three-group comparison using the Cox proportional hazards model with adjustment for age, sex, and the propensity scores in which carvedilol is treated as the reference group.

† Repeated pairwise comparison using the Cox proportional hazards model with adjustment for age, sex, and the propensity scores in which carvedilol is treated as the reference group.

‡ Repeated pairwise comparison using the Cox proportional hazards model with adjustment for age, sex, and stratified on quintiles of the propensity scores in which carvedilol is treated as the reference group.

***Supplementary Table 3. Beta-blockers prescribed in survivors of acute myocardial infarction*** in Taiwan

|  | **Year** | | | | | | | |  |
| --- | --- | --- | --- | --- | --- | --- | --- | --- | --- |
|  | **2003** | **2004** | **2005** | **2006** | **2007** | **2008** | **2009** | **2010** | **Total** |
| **Carvedilol, n**  **(%)** | 1307 (43.2) | 894 (47.0) | 293 (49.2) | 1257 (45.1) | 1137 (42.2) | 1101 (40.4) | 1049 (39.2) | 892 (34.1) | 7930 (41.7) |
| **Bisoprolol, n**  **(%)** | 500 (16.5) | 299 (15.7) | 125 (21.0) | 797 (28.6) | 906 (33.6) | 1011 (37.1) | 1147 (42.9) | 1290 (49.2) | 6075 (31.9) |
| **Propranolol, n**  **(%)** | 852 (28.1) | 492 (25.8) | 110 (18.5) | 496 (17.8) | 444 (16.5) | 427 (15.7) | 342 (12.8) | 311 (11.9) | 3474 (18.3) |
| **Atenolol, n**  **(%)** | 176 (5.8) | 94 (4.9) | 24 (4.0) | 105 (3.8) | 83 (3.1) | 80 (2.9) | 53 (2.0) | 46 (1.8) | 661 (3.5) |
| **Metoprolol, n**  **(%)** | 124 (4.1) | 86 (4.5) | 22 (3.7) | 96 (3.4) | 101 (3.8) | 89 (3.3) | 63 (2.4) | 65 (2.5) | 646 (3.4) |
| **Labetalol, n**  **(%)** | 26 (0.9) | 16 (0.8) | 13 (2.2) | 28 (1.0) | 14 (0.5) | 16 (0.6) | 14 (0.5) | 11 (0.4) | 138 (0.7) |
| **Others, n**  **(%)** | 43 (1.4) | 23 (1.2) | 8 (1.3) | 8 (0.3) | 11 (0.4) | 4 (0.2) | 6 (0.2) | 5 (0.2) | 108 (0.6) |
| **Total** | 3028 | 1904 | 595 | 2787 | 2696 | 2728 | 2674 | 2620 | 19032 |
